# Clinical and biological prognostic factors in follicular lymphoma patients

**DOI:** 10.1371/journal.pone.0272787

**Published:** 2022-08-04

**Authors:** Ádám Jóna, Anna Kenyeres, Sándor Barna, Árpád Illés, Zsófia Simon

**Affiliations:** 1 Faculty of Medicine, Department of Hematology, Medical School of Clinical Medicine, University of Debrecen, Debrecen, Hungary; 2 Doctoral School of Clinical Medicine, University of Debrecen, Debrecen, Hungary; 3 Scanomed Ltd., Debrecen, Hungary; Weill Cornell Medical College in Qatar, QATAR

## Abstract

**Introduction:**

Follicular lymphoma (FL) is an indolent, yet heterogeneous, B-cell lymphoproliferative disorder. Although most FL patients respond well to treatment, few with specific traits have a poor prognosis; the latter are difficult to define.

**Patients and methods:**

We retrospectively analyzed data from 143 FL patients treated at the University of Debrecen since 2009 and investigated prognostic factors that may influence the survival of FL patients.

**Results:**

A maximum standardized uptake value (SUVmax) cut-off of 9.85 at the staging positron emission tomography/computed tomography (PET/CT) (p = 0.0001, hazard ratio [HR]: 0.2535, 95% confidence interval [CI]: 0.1118–0.4878) and a lymphocyte/monocyte (Ly/Mo) ratio of 3.41 (p = 0.0027, HR: 2.997, 95% CI: 1.463–6.142), drawn at diagnosis, significantly predicted FL patients’ progression-free survival (PFS). A staging SUVmax >9.85 with Ly/Mo <3.41 could delineate a high-risk group of FL patients (p<0.0001, HR: 0.0957, 95% CI: 0.03416–0.2685). Similarly, a significant difference was shown with an SUVmax cut-off of 3.15 at the interim PET/CT (p<0.0001, HR: 0.1614, 95% CI: 0.06684–0.3897). A staging SUVmax >9.85 in conjunction with interim SUVmax >3.15 predicted poor prognosis (p<0.0001, HR: 0.1037, 95% CI: 0.03811–0.2824). The PFS difference was translated into overall survival (OS) advantage (p = 0.0506, HR: 0.1187, 95% CI: 0.01401–1.005).

**Conclusion:**

Biological prognostic factors, such as the Ly/Mo ratio, may improve the prognostic assessment of staging PET/CT. The survival advantage observed in PFS is translated into OS when determined using a combination of staging and interim SUVmax. We recommend investigating additional biological prognostic factors while highlighting the role of PET/CT in FL.

## Introduction

Follicular lymphoma (FL) is an indolent, germinal center B-cell–derived lymphoproliferative disease [1]. In general, FL is associated with the undue function of the proto-oncogene *BCL2*, which is activated by the translocation of t(14; 18) (q32; q21) [2]. FL is the most common indolent non-Hodgkin lymphoma (NHL) in the Western world [3], representing 35% of all NHLs.

FL is a biologically and clinically heterogeneous disease with wide variation in the outcomes of individual patients. The results of FL treatment have improved significantly; because of the introduction of the anti-CD20 antibody rituximab, the median overall survival (OS) of FL patients is approaching 20 years [4]. However, most patients eventually relapse. The ability to provide individualized treatment based on the risk assessment of individual patients is the subject of ongoing research.

Classically, histological grade, tumor mass, and Follicular Lymphoma International Prognostic Index (FLIPI) of 1 (involvement of >4 lymph node regions, elevated lactate dehydrogenase (LDH), >60 years of age, advanced stage of disease, and <120 g/L hemoglobin) and -2 (elevated beta-2 microglobulin, largest diameter lymph node >6 cm, bone marrow involvement, <120 g/L hemoglobin, >60 years of age) are the parameters that distinguish low- and high-risk patients [5]. One weakness of FLIPI is that it has been determined using retrospective data. The other is that it does not define a treatment indication. Moreover, like the International Prognostic Index, FLIPI only represents a few high-risk patients. Although a modified version, namely FLIPI2, was designed to overcome these issues, treatment is still determined based on high tumor mass according to the criteria of the GELF [6] or British National Lymphoma Investigation [7,8].

Several reports have confirmed the unfavorable survival of FL patients in whom the condition progresses early after treatment cessation; FL is expected to progress within 24 months in 20% of patients [9–11]. Unfortunately, such FL patients, who may have a poor prognosis, cannot be distinguished from the rest in advance. Hence, it is necessary to precisely predict patient outcomes.

## Patients and methods

We retrospectively investigated the prognostic factors of FL patients treated between May 2009 and June 2020 at the University of Debrecen, Department of Hematology. Factors that may have influenced survival include histology, age, stage, sex, staging-, interim- and restaging maximum standardized uptake value (SUVmax), presence or absence of B symptoms, bone marrow involvement, Eastern Cooperative Oncology Group (ECOG) performance status, hemoglobin, LDH, beta-2 microglobulin, absolute lymphocyte (Ly), and monocyte (Mo) count, and lymphocyte/monocyte (Ly/Mo) ratio. Progression of disease within 24 months (POD24) was calculated from the time of diagnosis until progression. The patients were informed consented before treatment initiation in written form to collect and publish their data retrospectively, according to the Declaration of Helsinki. The study did not included minors. This retrospective analysis was approved by the Regional and Institutional Research Ethics Committee of the University of Debrecen (DE RKEB/IKEB 5694–2021). The patients were treated according to current institutional guidelines. Briefly, the ‘watch and wait’ approach was used for patients not meeting the Groupe d’Etude des Lymphomes Folliculaires (GELF) [6] criteria. Rituximab monotherapy was used in older adults, and radiotherapy was used in the case of localized disease. Grade 1 and 2 patients received R-CVP (rituximab, cyclophosphamide, vincristine, prednisolone) until 2015, while bendamustine became widely available since 2015. Grade 3 patients received R-CHOP (cyclophosphamide, doxorubicin, vincristine, prednisolone) chemotherapy. Obinutuzumab has been administered to high-risk patients since 2018 or in clinical trials. The patients consented before treatment initiation to have their data collected and published retrospectively, according to the Declaration of Helsinki.

Positron emission tomography/computed tomography (PET/CT) has been routinely used as an imaging modality in the University of Debrecen since May 2009. However, it was not routinely performed in interim and restaging settings. Staging PET/CT was performed for every patient after a histological diagnosis of the disease, unless there was a clinical urgency, or the patient was treated primarily in another institution with no access to PET/CT scans. Interim PET/CT was performed after three cycles of immune-chemotherapy, and restaging PET/CT was done 4–6 weeks after completion of induction treatment.

All PET/CT examinations were based on a detailed institutional protocol described as follows. The fasting time before examination was 6 h. When the patient arrived at our center, we checked their blood glucose level and performed the test if it was <12.5 mmol/L. The patients’ weight and height were also measured. The radiopharmaceuticals were injected with a specific injector, Intego (Medrad-Bayer, Levercusen, Germany). The injected amount was based on the patient’s body weight: 4.4 MBq/kg. The accumulation time was 60 min for all cases, measured using a stopwatch. During the accumulation time, the patients were required to rest. They were not allowed to watch television or mobile phones and could not hear intensive or stressful music. They were required to drink 1 L of water before and during the waiting time. Before the examination, the patients had to empty their bladders. Five minutes before the end of the accumulation time, the patients laid down on the PET/CT scanner table (Philips Gemini ToF 64 (Amsterdam, Netherlands). First, we performed a CT overview called the CT localizer, and set the PET and CT ranges accordingly. Whole-body CT was performed, and when the stopwatch reached 60 min, PET was started. CT parameters were as follows: 200 mAs, 120 kV, 60/min rotation time, and one pitch. The reconstruction kernel was the abdomen, with a slice thickness of 5 mm. PET parameters were 2 min/bed position, usually 6–7 bed positions/patients. We used a 33% overlap. The correct Se PET range was based on the thigh. The reconstruction was performed on default PET time-of-flight reconstruction of the Philips Acquisition workspace. The SUV calibration was performed on every first day of the month, and the accepted results were <10% of the original value.

The SUVmax measurement was performed using the Interview Fusion version 3.03.077.0007 software (Mediso, Budapest), which has cross-validation with the Philips ISP system. The SUVmax calculation was based on body weight, and the SUV dimensions were g/mL. A nuclear medicine specialist measured the lesions on the fused images; we placed globus volume of interests onto the areas with the highest intensive uptake.

Histology was performed from the most accessible site because histology results are needed to order PET/CT imaging.

Patient outcomes were analyzed based on progression-free survival (PFS) and OS. PFS was calculated from diagnosis to June 2020, relapse or progression of disease, histological transformation, or death, whereas OS was calculated from diagnosis to June 2020 or death. Factors that could affect survival were evaluated using a univariate analysis. Continuous variables were transformed to discrete either by exceeding normal values or calculating data cut-off values based on the receiver operating characteristic (ROC) curves. State variables were defined as the events defined for PFS. A multivariable Cox regression model with the Enter method was used to obtain the hazard ratio (HR). Survival was estimated using the Kaplan–Meier method. Comparison of survival curves was based on the log-rank test. Statistical significance was set at p<0.05.

## Results

We investigated 143 FL patients with a median age of 54 years. A minority of these patients had B symptoms. Approximately two-thirds of the cases had grade 1 or 2 histology. Most patients were diagnosed with advanced-stage disease. POD24 was detected in 32 patients. We identified 114 accessible staging PET/CT scans, of which 64 had an interim and 80 had a restaging scan. The first-line treatment was dominated by the anti-CD20 antibody, rituximab, whereas the major chemotherapy backbone was -CHOP (-like), -CVP, or bendamustine **(Table 1).**

**Table 1:** Patient characteristics.

Patients	143
Female	80
Age (yrs, median, range)	54 (25–85)
Follow up (months, median, range)	54 (3–136)
B symptoms	55
Extranodal involvement	80
Bone marrow involvement	68
**Histology**	
Grade 1	43
Grade 2	48
Grade 3a	27
Grade 3b	7
FL ND	18
**Stage**	
I.	15
II.	14
III.	40
IV.	73
ND	1
Staging PET/CT cases	114
Interim PET/CT cases	64
Restaging PET/CT cases	80
**First line treatment**	** **
W&W	16
RT	3
R mono	2
R-CVP	16
R-CHOP (CEOP)	65
R-benda	34
G-benda	7

ND–not defined, W&W–watch and wait, RT–radiotherapy, R–rituximab, CVP—cyclophosphamide, vincristine, prednisolone, CHOP—cyclophosphamide, doxorubicin, vinristine, prednisolone, CEOP–cyclophosphamide, etoposide, vincristine, prednisolone, benda–bendamustin.

Univariate analysis showed that staging (cut-off: 9.85), interim (cut-off: 3.15), and restaging (2.68) SUVmax; LDH; Mo; and Ly/Mo ratio (cut-off: 3.41) affected PFS. Based on these results, we proposed two multivariate models using the Enter method. The first model used staging (cut-off: 9.85) and interim SUVmax (cut-off: 3.15); LDH; and Ly/Mo ratio (cut-off: 3.41); staging (cut-off: 9.85) and interim SUVmax (cut-off: 3.15) values remained as the independent prognostic factors for PFS in this model. The other model used staging SUVmax (cut-off: 9.85), Ly/Mo ratio (cut-off: 3.41), and LDH; SUVmax (cut-off: 9.85) and Ly/Mo ratio (cut-off: 3.41) emerged as the independent prognostic factors for PFS **(Table 2).**

**Table 2:** Univariate and multivariate analysis of prognostic factors that could possibly affect survival of follicular lymphoma patinets’.

		**Univariate analysis (PFS)**		
	Sig.	HR	95,0% CI for HR	
			Lower	Upper
Histology	0,929	6856,187	0,000	4,292E+87
Age >65 yrs	0,058	0,371	0,133	1,035
Gender	0,107	0,620	0,347	1,109
Staging SUVmax >9.85	**0,001**	**3,531**	**1,650**	**7,553**
Interim SUVmax >3.15	**0,0004**	**6,192**	**2,266**	**16,919**
Restaging SUVmax >2.68	**0,011**	**3,033**	**1,295**	**7,104**
Stage	0,233	2,488	0,557	11,119
B-symptoms	0,682	1,142	0,606	2,151
Bone marrow involvement	0,893	0,956	0,495	1,845
ECOG	0,255	2,382	0,534	10,625
Hbg <120 g/L	0,097	1,972	0,885	4,396
LDH >220 U/L	0,014	3,032	1,250	7,355
B2M >2.53 mg/L	0,822	1,099	0,485	2,491
Ly >3.10 G/L	0,230	1,797	0,690	4,682
Mo >0.9 G/L	**0,006**	**5,609**	**1,636**	**19,230**
Ly/Mo ratio <3.41	**0,005**	**2,963**	**1,395**	**6,295**
Extranodal involvement	0,923	1,040	0,470	2,302
	** **	**Multivariate analysis (Enter method) 1. (PFS)**	** **	** **
	Sig.	HR	95,0% CI for HR	
			Lower	Upper
LDH >220 U/L	0,776	0,840	0,252	2,798
Ly/Mo ratio <3.41	0,502	1,538	0,438	5,403
Interim SUVmax >3.15	**0,021**	**5,007**	**1,277**	**19,633**
Staging SUVmax >9.85	**0,036**	**5,609**	**1,124**	**27,992**
	** **	**Multivariate analysis (Enter method) 2. (PFS)**	** **	** **
	Sig.	HR	95,0% CI for HR	
			Lower	Upper
LDH >220 U/L	0,778	1,155	0,423	3,152
Ly/Mo ratio <3.41	**0,035**	**2,648**	**1,069**	**6,558**
Staging SUVmax >9.85	**0,008**	**3,929**	**1,438**	**10,735**

PFS–progression-free survival, SUVmax–maximum of standardized uptake value, ECOG—Eastern Cooperative Oncology Group, Hgb–hemoglobin, B2M - beta-2 microglobulin, Ly–lymphocyte, Mo–monocyte, LDH–lactate dehydrogenase, Sig.–significance, HR–hazard ratio, CI–confidence interval.

**Fig 1:**
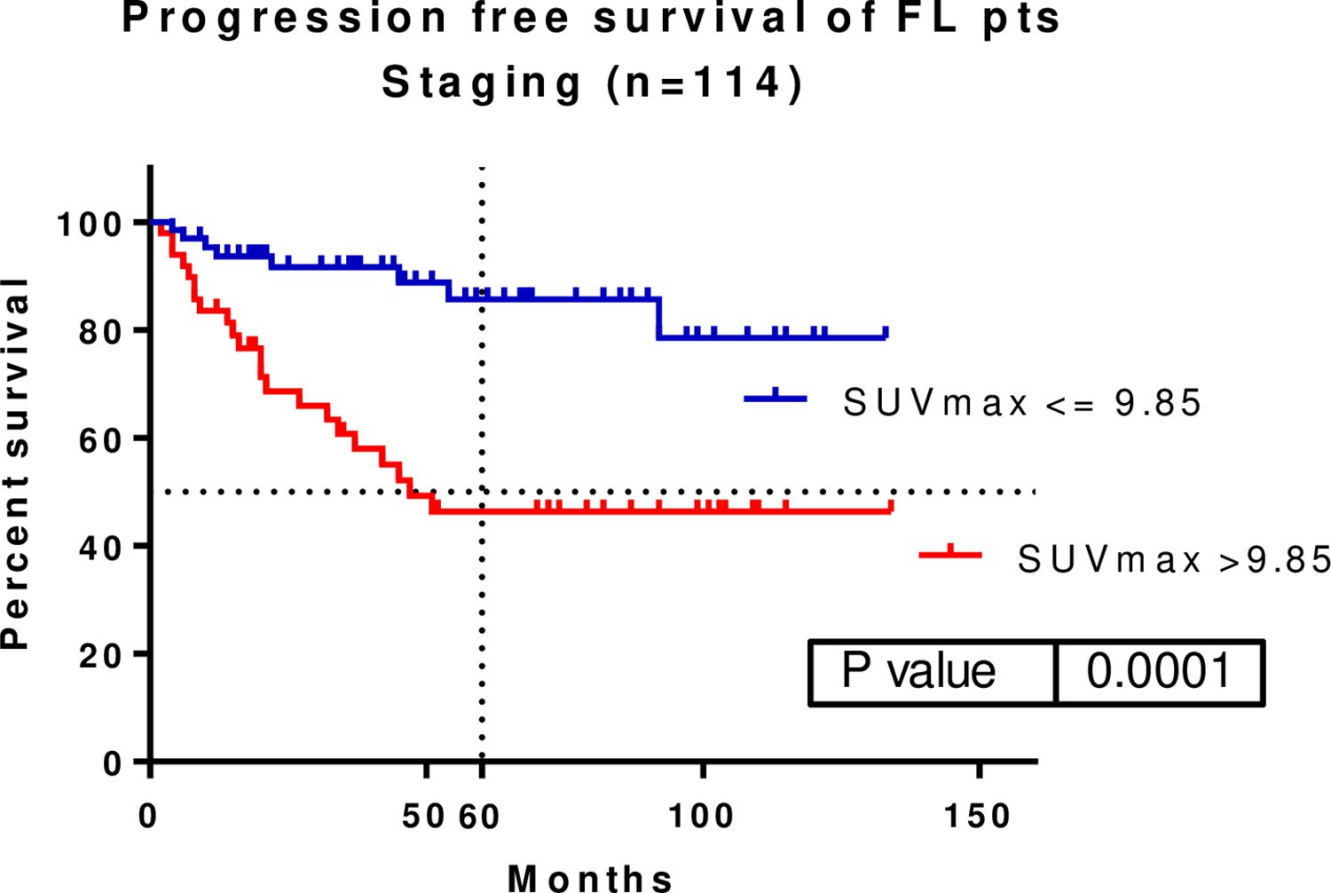
We found a SUVmax cut-off value of 9.85 at the staging PET/CT to significantly separate follicular (FL) patients’ progression-free survival (PFS) (p = 0.0001, HR: 0.2535, 95%CI: 0.1118–0.4878). Five-year PFS was 85.63 vs. 46.36%. SUVmax–maximum of standardized uptake value.

A total of 114 patients underwent PET/CT as a staging imaging modality since May 2009. An ROC curve of the SUVmax showed that a cut-off value of 9.85 could predict the patients’ survival significantly. When illustrating the patients’ PFS, a significant difference was found when using the defined SUVmax cut-off value (p = 0.0001, HR: 0.2535, 95% CI: 0.1118–0.4878). The median PFS of FL patients with SUVmax >9.85 was 47 months, whereas median PFS with SUVmax ≤9.85 was not achieved. The five-year PFS rates of FL patients with staging SUVmax >9.85 and ≤9.85 were 85.63% and 46.36%, respectively. There was no significant difference in the patients’ OS (five-year OS: 96.92% vs. 85.09%) (**Fig 1).**

**Fig 2:**
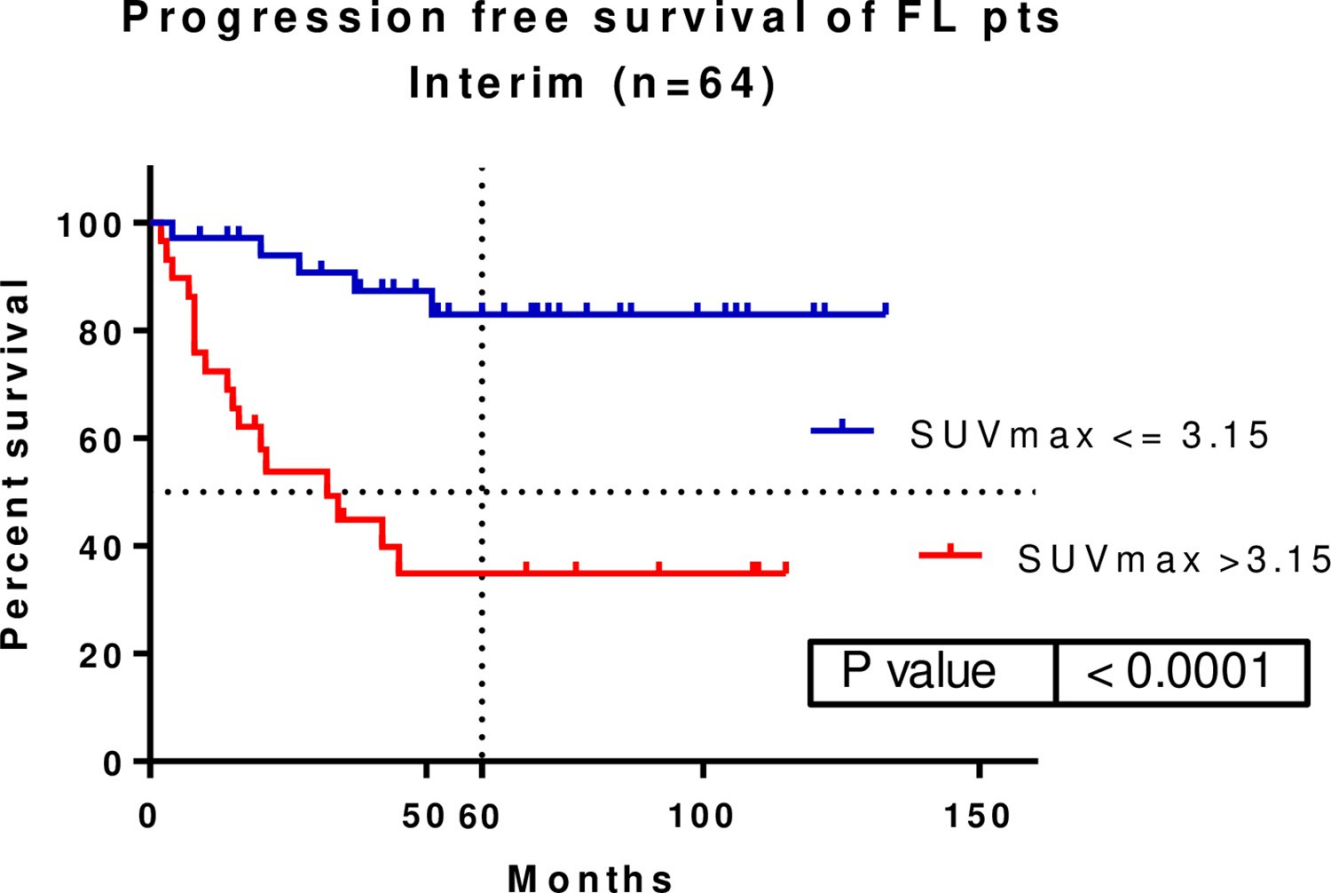
A significant progression-free survival (PFS) difference was shown with a SUVmax cut-off of 3.15 at the interim PET/CT (p<0.0001, HR: 0.1614, 95%CI: 0.06684–0.3897). Five-year PFS was 82.94 vs. 34.86%. SUVmax–maximum of standardized uptake value.

A total of 64 patients underwent interim PET/CT scans. An SUVmax of 3.15, was identified as the cut-off by the ROC curve. A significant difference was found when illustrating the patients’ PFS when using the defined SUVmax cut-off value (p<0.0001, HR: 0.1614, 95% CI: 0.06684–0.3897). The median survival was 32 months for FL patients with SUVmax >3.15 in the interim PET/CT. Median survival of patients with interim SUVmax ≤3.15 was not met. The five-year PFS rates of patients with interim SUVmax >3.15 and ≤3.15 were 82.94% and 34.86%, respectively **(Fig 2).** The OS did not significantly differ among these patients (5-year OS: 95.00 vs. 86.16%).

**Fig 3:**
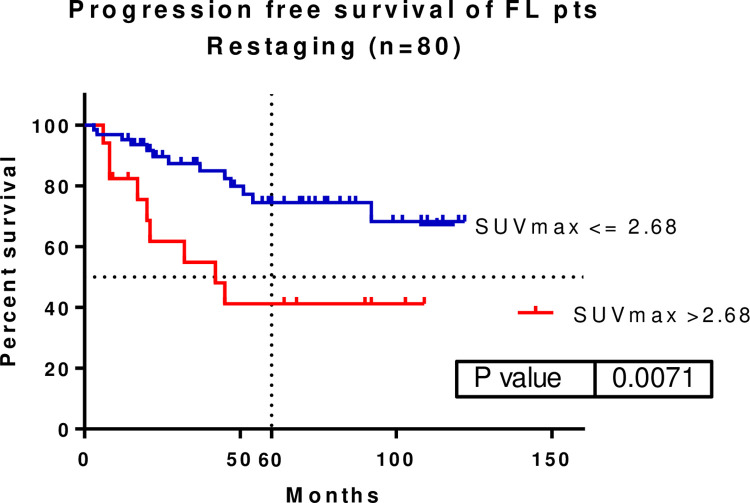
A significant progression-free survival (PFS) difference was shown with a SUVmax cut-off of 2.68 at the restaging PET/CT (p = 0.0071, HR: 0.2276, 95%CI: 0.07753–0.6683). Five-year PFS was 74.48 vs. 41.17% SUVmax–maximum of standardized uptake value.

In total, 80 patients underwent restaging PET/CT scans. An SUVmax of 2.68 was identified as the cut-off by the ROC curve. A significant difference was found when illustrating the patients’ PFS when using the defined SUVmax cut-off value (p = 0.0071, HR: 0.2276, 95% CI: 0.07753–0.6683). The median survival was 42 months for FL patients with restaging SUVmax >2.68. Median survival was not achieved in patients with restaging SUVmax ≤2.68. The five-year PFS rates for patients with restaging SUVmax >2.68 and ≤2.68 were 74.48% and 41.17%, respectively **(Fig 3.).** There was no significant difference in the OS of these patients (5-year OS: 92.52 vs. 81.81%).

**Fig 4:**
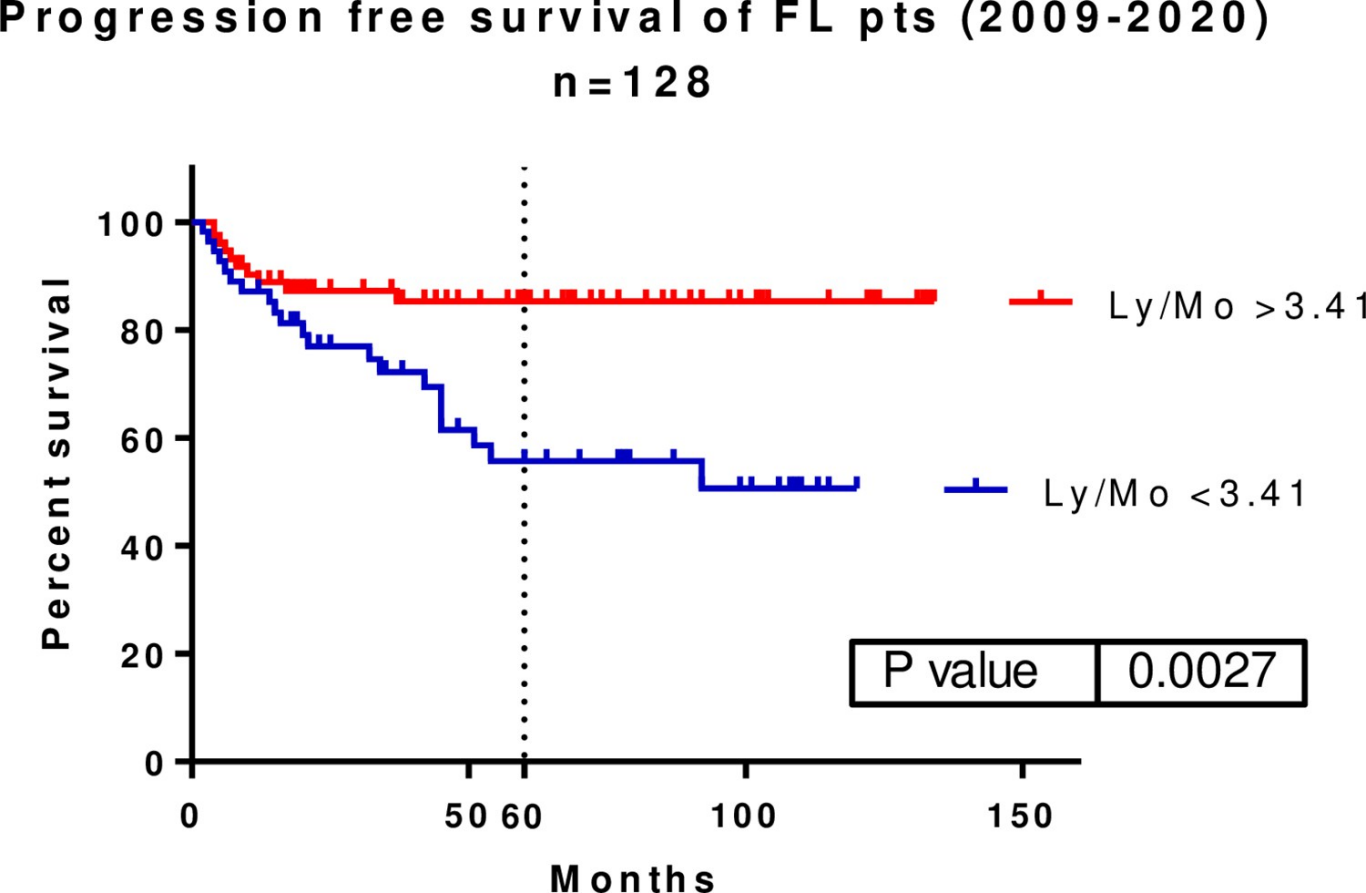
Lymphocyte/ monocyte (Ly/Mo) ratio of 3.41 drawn at diagnosis also significantly predicted PFS (p = 0.0027, HR: 2.997, 95% CI: 1.463–6.142). Five-year PFS was 85.30 vs. 55.73%. PFS–progression-free survival, Ly–lymphocyte, Mo–monocyte.

A total of 128 patients had accessible blood counts in the medical record system. The cut-off value for Ly/Mo was found to be 3.41 with the ROC curve. When illustrating the PFS of these patients using the defined value, we found a statistically significant inter-group difference (p = 0.0027, HR: 2.997, 95% CI: 1.463–6.142). The median PFS was not met in any subgroup. The five-year PFS rates were 85.30% and 55.73% in patients with Ly/Mo >3.41 and <3.41, respectively **(Fig 4).** The OS did not significantly differ among these patients (5-year OS: 95.00 vs. 86.28%).

**Fig 5:**
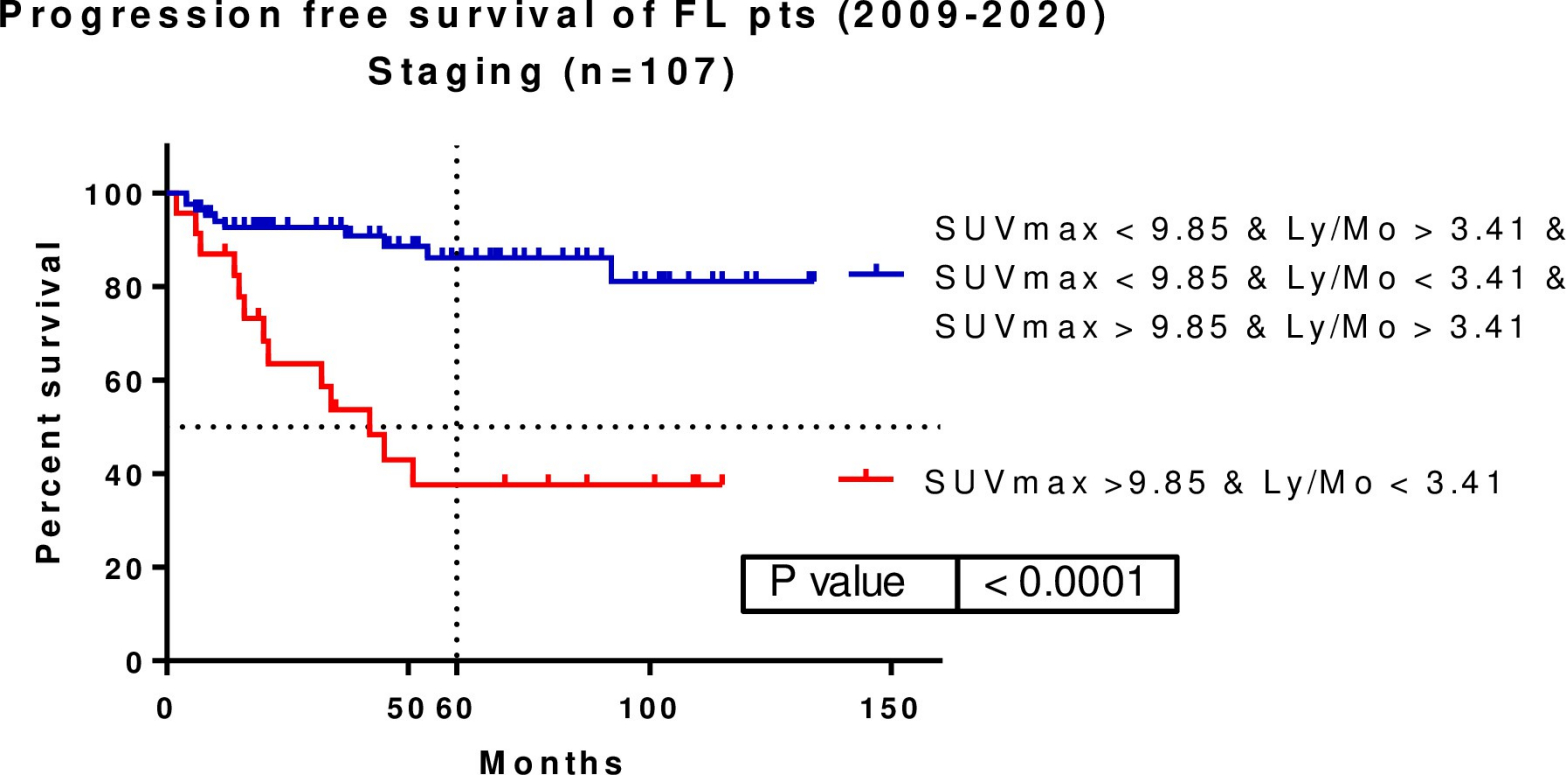
Combining patients with staging SUVmax >9.85 and Ly/Mo < 3.41 a high-risk group of FL patients can be identified (p<0.0001, HR: 0.0957, 95%CI: 0.03416–0.2685). Five-year PFS was 86.16 vs. 37.59%. SUVmax–maximum of standardized uptake value, Ly–lymphocyte, Mo–monocyte.

FL patients with staging SUVmax >9.85 and Ly/Mo <3.41 comprised a high-risk group (p<0.0001, HR: 0.0957, 95% CI: 0.03416–0.2685). The median survival was 42 months in these patients, whereas median survival was not achieved among the remaining patients. The five-year PFS rates for the high-risk group and the remaining patients were 86.16% and 37.59%, respectively **(Fig 5).**

**Fig 6:**
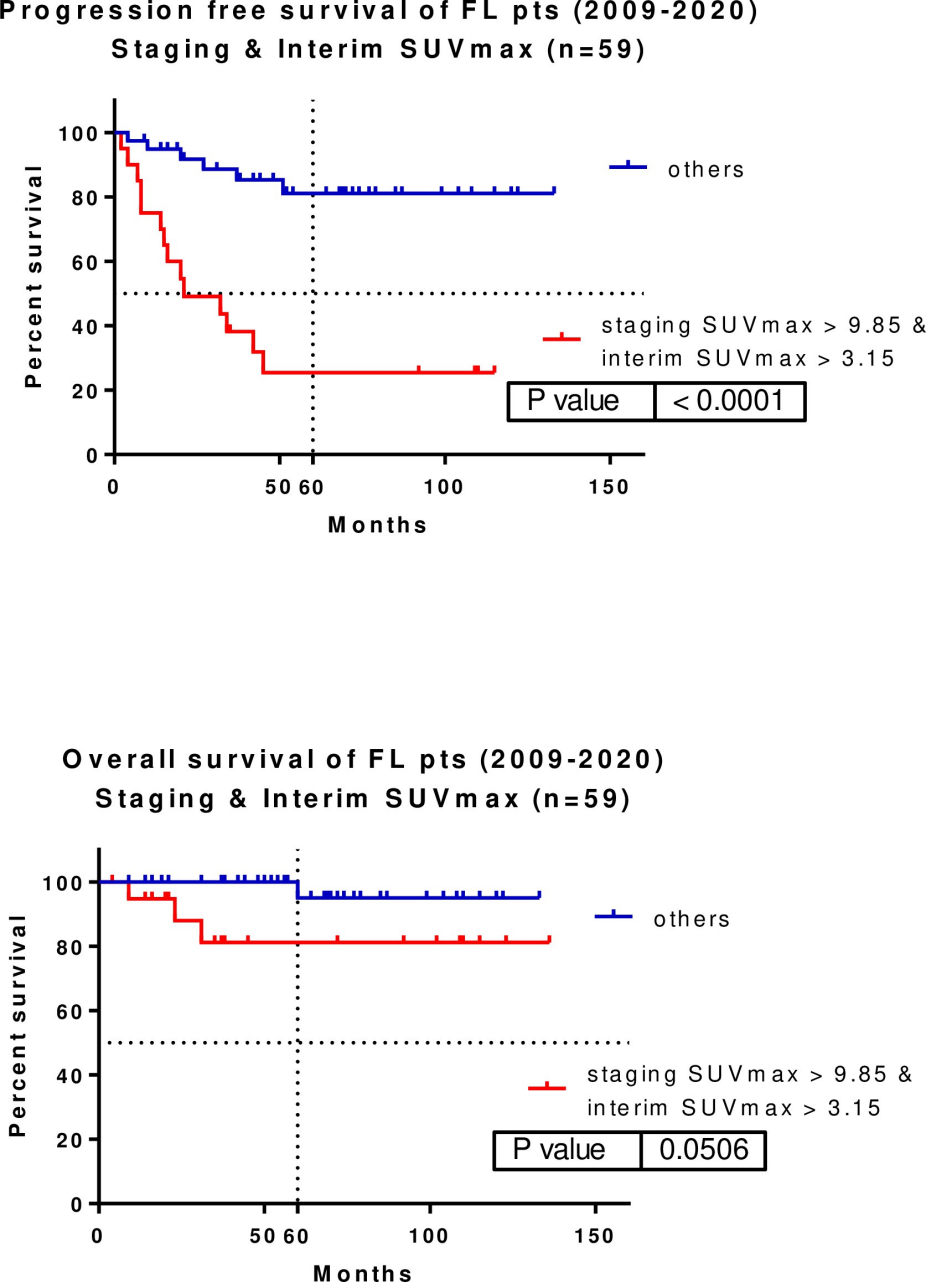
Combining patients with staging SUVmax >9.85 and interim SUVmax >3.15, a high-risk group of FL patients can be identified (p<0.0001, HR: 0.1037, 95%CI: 0.03811–0.2824). Five-year PFS was 81.035 vs. 25.45%. The PFS difference is translated into OS disadvantage (p = 0.0506, HR: 0.1187, 95%CI: 0.01401–1.005). Five-year OS was 95.00 vs. 81.20%. SUVmax–maximum of standardized uptake value.

Patients who had a staging SUVmax >9.85 and interim SUVmax >3.15 could also be identified as high-risk (p<0.0001, HR: 0.1037, 95% CI: 0.03811–0.2824). The median PFS of this group was 21 months, whereas median survival was not reached among the remaining patients. The five-year PFS rate in the high-risk group with staging SUVmax >9.85 and interim SUVmax >3.15 was 81.035%, whereas that in the remaining patients was 25.45% **(Fig 6)**. The difference in PFS was translated into OS disadvantage (p = 0.0506, HR: 0.1187, 95% CI: 0.01401–1.005). The five-year OS rates for this high-risk group and the remaining patients were 95.00% and 81.20%, respectively.

## Discussion

The initial total metabolic tumor volume (TMTV) measured using PET/CT strongly correlates with survival in FL patients who receive R-CHOP without maintenance treatment. Moreover, patients with TMTV >510 cm^3^ tend to have a significantly less favorable (33%) 5-year PFS than do patients with TMTV <510 cm^3^ (65%). The 5-year OS rates in the former and latter groups are 85% and 95%, respectively [12]. Our findings from the data of 114 FL patients showed that staging SUVmax predicted PFS when 9.85 was used as the cut-off. The survival advantage, however, did not translate into significant differences in OS. The 5-year PFS (85.29% vs. 48.25%) and OS (96.87 vs. 85.73%) rates predicted with the staging SUVmax were similar to those found with TMTV.

PET/CT is a standard imaging method for response evaluation in FDG-avid lymphomas including FL. However, the prognostic evaluation of FL by PET/CT is not widely established. In a study published in 2019, a survey of 33 FL patients found that interim PET/CT performed after three or four cycles of first-line treatment was predictive of PFS [13]. A meta-analysis published in 2016 found one trial that reported a positive correlation between positive or negative interim PET/CT results and PFS, while two studies reported a negative correlation [14]. Our results confirmed a positive correlation. The interim PET/CT scan of 64 FL patients, performed after three cycles of immunochemotherapy, and with a cut-off of 3.15 for SUVmax showed significant PFS survival benefit. However, the difference was not significant in terms of OS, which may be explained by the extent and efficacy of the treatment options and frequency of relapse [15]. A recent paper, published in 2019, reported 84 FL patients, of whom 59 and 24 underwent a baseline and an interim PET/CT scan, respectively. Similar to our results, they found a positive correlation between the baseline SUVmax of 10.44 and PFS. However, the difference in survival was not significant in terms of OS. Interim PET/CT results interpreted as “positive /negative,” “Deauville score 1–3 and 4–5,” and “ΔSUVmax (change of SUVmax from baseline to interim point)” were neither prognostic for PFS nor OS [16].

Nevertheless, when we grouped together patients with staging and interim SUVmax below the cut-off, we could identify a patient group with a significantly poor prognosis. The significant survival disadvantage in PFS was translated into a difference, albeit non-significant, in OS. The condition of half of these high-risk group patients progressed within 21 months, determining POD24 patients after three cycles (and practically three months) of treatment. POD24 also translates into an OS disadvantage. If our findings are confirmed in further prospective trials with larger samples, patients belonging to the high-risk group, as determined by unfavorable interim PET/CT scan results, may require a more aggressive therapeutic approach than that usually undertaken for FL owing to its indolent nature. It would be fortunate to predict these adverse cases even earlier, possibly at the time of diagnosis. This could also allow clinicians to determine whether a patient with a good prognosis could receive therapy with a “permissive” approach. For example, clinicians could identify which patients should discontinue maintenance therapy during the ongoing coronavirus disease pandemic to moderate B-cell depletion [17] and which patients require continuous granulocyte colony stimulating factor support.

Several reports show that PET/CT results at restaging predict PFS. PET/CT performed three months after the completion of induction treatment is also an independent prognostic factor [18,19]. A meta-analysis of large multicenter trials verified in 2014 that a negative PET/CT performed after six cycles of induction treatment was prognostic for both PFS and OS [20]. Our results of restaging PET/CT scans were also prognostic for PFS at a cut-off of 2.68. However, the results were not significant in terms of OS.

A low Ly count is an adverse prognostic factor not only in Hodgkin lymphoma (HL) [21] but also in FL [22], and may be related to the patient’s immunity. In contrast, the Mo count could be related to the tumor microenvironment [23]. An elevated Mo count is associated with a poor prognosis. The Ly/Mo ratio has also been reported as a prognostic factor in HL [24,25]. In our multivariate analysis, Ly/Mo ratio emerged as a prognostic factor for our sample. The cut-off value of 3.41, which was similar to that found in our HL population [26] and in Italian [27] and Hong Kong-based FL datasets [28]. The Ly/Mo ratio is not standardized because the results are heterogeneous; however, when the Ly/Mo ratio is combined with the staging SUVmax, a patient group with a significantly poor prognosis can be identified. Further, larger studies are warranted to determine the prognostic value of the Ly/Mo ratio in FL.

There is a need to precisely predict the treatment outcomes in advance for patients with FL. Yet, it is difficult to identify patients with FL who may have a poor prognosis at early stages of treatment. We found that the prognostic value of early PET/CT findings could be improved by combining them either with biological factor data, such as the lymphocyte/monocyte ratio or with the PET/CT findings from interim stages of evaluation. Clinicians who treat and manage patients with FL could use our findings to identify patients at high-risk of unfavourable outcomes.

## Conclusion

We infer that biological prognostic factors, such as the Ly/Mo ratio, are essential because they may improve the prognostic assessment of staging PET/CT. Further, by combining staging and interim SUVmax and grouping patients as per the cut-offs, we could identify a difference in the overall survival. Our findings show that the combined use of staging and interim SUVmax could have better prognostic value in FL than using only either of the two values. Therefore, we consider it necessary to investigate additional biological prognostic factors while highlighting the role of PET/CT in the diagnosis and treatment of FL patients.

## Supporting information

S1 Raw data This file contains raw data of the anonymized patients the study was conducted based on. Certficate of editing DMJNA_2_movm-a57z6.pdf This document certifies that the paper has been edited to ensure that the language is clear and free of errors. The logical presentation of ideas and the structure of the paper were also checked during the editing process. The edit was performed by professional editors at Editage, a division of Cactus Communications, in cooperation with Taylor & Francis Group. The intent of the author’s message was not altered in any way during the editing process. The quality of the edit has been guaranteed, with the assumption that our suggested changes have been accepted and have not been further altered without the knowledge of our editors.
(XLSX)

## Abbreviation

CHOP: cyclophosphamide, doxorubicin, vincristine, prednisolone
CI: confidence interval
CVP: cyclophosphamide, vincristine, prednisolone
ECOG: Eastern Cooperative Oncology Group
FL: follicular lymphoma
FLIPI: Follicular Lymphoma International Prognostic Index
GELF: Groupe d’Etude des Lymphomes Folliculaires
HR: hazard ratio
LDH: lactate dehydrogenase
Ly: lymphocyte
Mo: monocyte
NHLs: non-Hodgkin’s lymphomas
OS: overall survival
PET/CT: positron emission tomography/computed tomography
PFS: progression-free survival
POD24: Progression of disease within 24 months
ROC: receiver operating characteristic
SUVmax: maximum standardized uptake value[s]
TMTV: total metabolic tumor volume
